# Scratching the Surface: Re-evaluating the Management of Corneal Abrasions

**DOI:** 10.7759/cureus.103351

**Published:** 2026-02-10

**Authors:** Caitlyn M Cooper, Erich Berg, John Ashurst

**Affiliations:** 1 Research, Midwestern University Arizona College of Osteopathic Medicine, Glendale, USA; 2 Ophthalmology, University of Louisville, Louisville, USA

**Keywords:** corneal abrasion, corneal epithelial healing, emergency ophthalmology, ocular trauma, topical anesthetics

## Abstract

This narrative review examines the current evidence on the pathophysiology, clinical evaluation, and management of corneal abrasions, while highlighting areas of variability and ongoing controversy in clinical practice.

Corneal abrasions are among the most common causes of ocular pain presenting to emergency departments. Although often self-limited, delayed diagnosis or inappropriate management can result in serious complications, including infectious keratitis, corneal ulceration, and permanent vision loss. These injuries are managed by multiple healthcare specialties, including emergency medicine, primary care, optometry, and ophthalmology, which may contribute to the variability in treatment approaches and the lack of standardized, evidence-based protocols.

Diagnosis is primarily clinical and relies on careful history, visual acuity assessment, fluorescein staining, and exclusion of penetrating injury or infection. Management options include topical antibiotics, oral and topical analgesics, nonsteroidal anti-inflammatory drugs (NSAIDs), cycloplegics, bandage contact lenses, and supportive care. However, many commonly used interventions lack robust prospective evidence, and practice patterns vary widely.

A key area of controversy involves the outpatient use of topical anesthetic agents for pain control. While limited short-term use has been proposed in selected settings, concerns persist regarding epithelial toxicity, adherence, and patient safety. This review synthesizes perspectives across specialties to delineate areas of agreement and uncertainty, highlighting the need for high-quality clinical studies with standardized outcomes to inform evidence-based management of corneal abrasions.

## Introduction and background

Each year, there are an estimated 2.5 million Emergency Department (ED) visits in the United States related to ocular complaints, which may be broadly categorized as non-traumatic, such as conjunctivitis or spontaneous subconjunctival hemorrhage, or traumatic, including corneal abrasions, corneal foreign bodies, and globe rupture [1]. Among traumatic eye conditions, corneal abrasions are the most common cause of eye pain presenting to the ED, accounting for approximately 357,000 visits annually [2]. Although often considered minor injuries, corneal abrasions can result in serious complications, including infectious keratitis, corneal ulceration, corneal scarring, and permanent vision loss if diagnosis is delayed or management is inappropriate [3]. Despite their high incidence, substantial variability exists in the evaluation and treatment of corneal abrasions across clinical settings [2]. Because these injuries are encountered by multiple healthcare specialties, including emergency medicine, primary care, optometry, and ophthalmology, familiarity with current diagnostic and management considerations is essential to optimize patient outcomes.

Etiologies 

Corneal abrasions most commonly occur following superficial trauma to the corneal epithelium. Common mechanisms include mechanical injury from fingernails, makeup brushes, plant material, or foreign bodies, as well as chemical exposure and contact lens use [3]. In some cases, the presence of particulate matter such as sand or dirt, or severe ocular surface dryness, may predispose the cornea to epithelial disruption during eye rubbing [3].

Contact lens use represents a major risk factor for corneal abrasion. Improper lens handling, use of damaged or contaminated lenses, and extended or overnight wear can result in microscopic epithelial defects [3]. Overnight or extended use of contact lenses increases the risk of microbial keratitis by five- to 10-fold or more [4]. Micro-abrasions in contact lens users facilitate bacterial adherence and invasion, most commonly by *Pseudomonas aeruginosa*, which can lead to corneal ulceration, stromal melting, or perforation [5]. *P. aeruginosa* is known to survive in contact lens storage cases and demonstrates resistance to many standard disinfecting solutions [6]. In addition to bacterial infection, contact lens exposure to water during showering or swimming increases the risk of *Acanthamoeba* keratitis. Acanthamoeba is a free-living amoeba found in soil, dust, air, and freshwater and is highly resistant to chlorine-based disinfectants [6]. Microbial keratitis represents an ophthalmic emergency and is characterized by a painful epithelial defect with an associated corneal infiltrate, requiring urgent treatment to preserve vision [7]. Given the elevated risk of rapidly progressive infection in contact lens-associated abrasions, these patients are typically managed more aggressively in the ED, with empiric topical antibiotics that provide antipseudomonal coverage and a low threshold for urgent ophthalmology follow-up [7]. Delayed or inadequate treatment in this population increases the risk of corneal ulceration and permanent vision loss [7].

Non-traumatic corneal abrasions may also occur, most commonly in the setting of recurrent corneal erosion (RCE), a condition characterized by spontaneous breakdown of the corneal epithelium [8]. RCE most commonly affects adults between 30 and 80 years of age, with the highest prevalence reported in the third and fourth decades of life [8]. Risk factors include prior corneal trauma, epithelial basement membrane dystrophy (EBMD), prior ocular surgery, and comorbid conditions such as dry eye disease, diabetes mellitus, blepharitis, or ocular rosacea [8]. Prior corneal trauma accounts for approximately 45-64% of cases, while EBMD represents the second most common etiology, reported in 19%-29% of patients [8]. RCE typically presents with a sudden onset of eye pain, often upon awakening, without a clear history of trauma [9]. Incomplete eyelid closure during sleep or impaired corneal protection may contribute to epithelial injury in these cases. In hospitalized patients, perioperative corneal abrasions have been associated with inadequate eyelid protection during general anesthesia, particularly when Bell's phenomenon is suppressed [9]. 

Epidemiology

Corneal abrasions occur in approximately 3 per 1,000 ED visits [10]. Analysis of national ED data from 2010 to 2018 demonstrated that 31.6% of ophthalmic ED visits were trauma-related, with superficial corneal injury representing the most common traumatic diagnosis (13.9%) [1].

Workplace injuries account for up to 25% of ocular trauma [11]. A review of U.S. Bureau of Labor Statistics data from 2011 to 2020 found that work-related eye injuries most frequently occurred in men, individuals aged 25 to 34 years, and those employed in service, production, and installation or maintenance occupations [12]. The median number of missed workdays associated with these injuries was two [12]. Most injuries resulted from contact with objects or equipment, including foreign bodies entering or abrading the eye [12].

Contact lens use is another major contributor to corneal abrasions. Approximately one in six adults in the United States wears contact lenses, and more than 10% of corneal abrasions presenting to the ED are contact-lens related [3]. Poor lens hygiene practices are common and significantly increase the risk of ocular infection. In a national survey conducted by the Centers for Disease Control and Prevention, nearly 99% of contact lens users reported engaging in at least one behavior associated with increased infection risk [13]. These behaviors included overnight lens wear, extending replacement schedules, *topping off* disinfecting solution (adding new solution to old solution in the lens case rather than fully replacing it), and exposing lenses to freshwater sources, including tap water during showering, and swimming in pools, lakes, rivers, or hot tubs [13]. Even contact lenses approved for extended wear carry an increased risk of infection when worn overnight. Contact-lens associated hypoxia during sleep reduces oxygen delivery to the cornea and facilitates bacterial adherence, particularly by* P. aeruginosa *[14]. Consequently, contact lens use remains the single greatest factor for microbial keratitis [13].

RCE is most commonly associated with prior corneal trauma, accounting for 45%-64% of cases, followed by EBMD in 19%-29% of patients [15]. The condition most frequently affects adults between 30 and 40 years of age [8]. Epidemiologic data suggest that RCE is likely underreported, as mild episodes may resolve before clinical evaluation [15].

## Review

Pathophysiology

Corneal Functions and Characteristics

The cornea plays a central role in vision as the primary refractive surface of the eye, contributing approximately two-thirds of its total refractive power [16,17]. As the most anterior structure of the globe, it also serves as a protective barrier against environmental trauma and microbial invasion [16]. The cornea is transparent, avascular, and immune-privileged, and its dense sensory innervation explains the severe pain associated with even minor injuries [16].

Corneal thickness gradually increases from the central to the peripheral cornea and decreases with age [16]. Structurally, the cornea consists of both cellular and acellular components. The cellular elements include epithelial cells, keratocytes, and endothelial cells, while the acellular matrix is composed primarily of collagen and glycosaminoglycans [17]. From anterior to posterior, the cornea is organized into five layers: epithelium, Bowman’s layer, stroma, Descemet’s membrane, and endothelium [17].

Corneal Epithelium

A corneal abrasion is defined as a defect in the corneal epithelium, the most superficial layer of the cornea [3]. The epithelium is approximately 50 microns thick and composed of four to six layers of non-keratinized stratified squamous epithelial cells [3]. These layers include superficial cells, wing cells, and basal cells [3].

Superficial epithelial cells form tight junctions and interface directly with the tear film, maintaining a smooth optical surface [3]. Wing cells occupy the intermediate layers, while basal cells anchor the epithelium to the basement membrane via hemidesmosomes, providing structural stability [16]. Disruption of this epithelial-basement membrane interface may result in recurrent erosions or non-healing epithelial defects, such as those observed in EBMD [17].

Most corneal abrasions are limited to the superficial epithelium and heal within two to three days [17]. Injuries that extend to the basement membrane initiate a more complex wound-healing response and may result in prolonged healing or stromal scarring lasting several weeks [17].

Corneal epithelial cells have a lifespan of approximately seven to ten days and are continuously regenerated from limbal stem cells (LSCs) located at the corneoscleral junction [18]. These stem cells divide into transient amplifying cells that migrate centrally and differentiate into mature epithelial cells [19]. Homeostasis is maintained by balancing cell proliferation, centripetal migration, and surface cell loss [18,20]. Disruption of this process, as seen in LSC deficiency, results in persistent epithelial defects, stromal thinning, conjunctivalization, and corneal neovascularization [18].

Corneal Wound Healing

Corneal wound healing is essential for restoring epithelial integrity while preserving corneal transparency and refractive function [21]. Healing involves coordinated cellular migration, proliferation, adhesion, and differentiation, mediated by growth factors, cytokines, and extracellular matrix signaling at the site of injury [21]. The size and depth of the abrasion influence both the duration and complexity of the healing response [22].

Epithelial healing occurs in distinct but overlapping phases [23]. Following an initial lag phase marked by cellular reorganization and apoptosis, epithelial repair proceeds through cell migration, proliferation, and stratification, and re-establishment of adhesion complexes [23]. During migration, basal and suprabasal cells flatten and move as a cohesive sheet over the defect [23]. Subsequent proliferation replenishes epithelial cell numbers, followed by reassembly of hemidesmosomes and extracellular matrix attachments [23].

Immune cells also contribute to epithelial repair. Neutrophils are among the first responders, clearing cellular debris and releasing mediators that promote epithelial proliferation and corneal nerve regeneration [24]. Platelets provide additional inflammatory and reparative factors that support tissue remodeling [24]. Restoration of epithelial thickness and adhesion is critical for re-establishing corneal barrier function [24].

Emerging evidence suggests that circadian rhythms influence corneal wound healing [24,25]. Experimental studies demonstrate faster epithelial closure and increased mitotic activity when corneal abrasions occur in the morning compared to later in the day [24,25]. These findings highlight a potential role for circadian-regulated mechanisms in epithelial repair, though clinical implications remain under investigation.

Diagnosis

History and Physical Exam

Corneal abrasions are common but may be overlooked if a thorough history and examination are not performed. They should be considered in patients presenting with eye pain, foreign body sensation, photophobia, tearing, difficulty opening the eye, redness, or decreased visual acuity, particularly when recent trauma or contact lens use is reported [11]. Important historical elements include contact lens habits, occupational and recreational exposures that increase the risk of ocular injury, lack of protective eyewear, and activities preceding symptom onset [22].

External inspection using a penlight should precede fluorescein staining to identify eyelid abnormalities, foreign material, or signs of thermal or chemical injury [26]. If pain limits cooperation, a single dose of topical anesthetic may be used to facilitate examination [26]. Improvement in discomfort after anesthetic instillation may suggest a superficial corneal or conjunctival source of pain; however, this response is not specific and does not reliably exclude intraocular pathology (e.g., uveitis or acute angle-closure glaucoma) [22]. Topical anesthetics are intended for diagnostic use only, as their prolonged or unsupervised use has been associated with delayed epithelial healing, toxic keratopathy, and permanent corneal damage [27].

Examination findings that raise concern for penetrating injury or infection include an irregular or peaked pupil, corneal infiltrates, hyphema, hypopyon, or marked vision loss, all of which warrant urgent ophthalmologic consultation [26]. In uncomplicated abrasions, the pupil is typically round and reactive, with conjunctival injection and localized epithelial disruption [26].

Visual acuity should be assessed early, and is often done immediately following inspection [26]. Abrasions involving the visual axis may result in subjective vision loss, and a decrease of two or more lines from a patient's baseline visual acuity should prompt urgent ophthalmologic evaluation [26]. Extraocular movements should be intact; pain with movement or restricted motility suggests deeper orbital pathology [26]. Fluorescein staining confirms the diagnosis and reveals the size and pattern of epithelial defects [3]. Abrasions appear yellow under white light and fluoresce green under cobalt-blue illumination. Linear or punctate staining may suggest a retained foreign body, while branching patterns raise concern for herpetic disease and warrant referral [26]. While handheld devices such as a Wood’s lamp may be used in resource-limited or bedside settings, slit-lamp biomicroscopy is the preferred examination modality, as it provides superior magnification and sensitivity and may detect small or subtle epithelial defects that can be missed with handheld examination. In one prospective study, a Wood’s lamp detected only about 52% of corneal abrasions compared with the slit-lamp exam [28]. Eyelid eversion is essential to identify hidden foreign material [26].

Treatment

Most corneal abrasions heal within two to three days, but inappropriate management may lead to infection, scarring, or vision loss [11]. Initial management is often undertaken by emergency medicine and primary care clinicians and may include topical antibiotics, oral or topical analgesics, cycloplegics, and supportive care. Interventions such as bandage contact lens (BCL) placement are typically reserved for ophthalmologists or optometrists with appropriate expertise and follow-up capability. Few interventions have been evaluated in large prospective trials, and many recommendations are based on clinical experience and consensus, contributing to variability in practice.

Antibiotics

Topical antibiotic prophylaxis is commonly prescribed despite limited evidence supporting its routine use in uncomplicated abrasions [29]. Current recommendations favor antibiotics in patients with risk factors for microbial keratitis, including contact lens use and traumatic exposure to contaminated material [30]. Ointments such as erythromycin are often used in non-contact lens wearers due to their lubricating properties, though antibiotic drops may be preferred by some patients because of easier administration and less visual blur. Both antibiotic drops and ointments are acceptable for corneal abrasions, with no strong evidence favoring one formulation over the other; selection is typically guided by patient comfort, ease of administration, and clinical context [26,30]. Fluoroquinolones are preferred for contact lens-associated abrasions to provide Pseudomonas coverage [26,31]. Antibiotics are typically prescribed four times daily for 3-5 days or until epithelial healing is confirmed [26,30].

Randomized trials and systematic reviews have not demonstrated clear benefits of prophylactic antibiotics in accelerating healing or preventing infection in low-risk cases [30,32,33]. Nonetheless, antibiotics remain widely used given the potential severity of infectious complications.

Pain Management

Pain control is an important component of treatment. Oral analgesics such as acetaminophen and NSAIDs are often sufficient [26]. While opioid analgesics might be used in select cases of severe pain, evidence supporting additional benefit is limited, and they are not routinely included in standard treatment recommendations [26].

Topical NSAIDs

Topical nonsteroidal anti-inflammatory drugs (NSAIDs) have demonstrated modest but consistent reductions in pain and oral analgesic use during the first 48 hours following abrasion, without increased complication rates [34]. A recent systematic review evaluating topical analgesic therapies for corneal abrasion found that topical NSAIDs were the only intervention associated with statistically significant reductions in patient-reported pain scores and decreased use of oral analgesics [34]. Importantly, their use was not associated with an increased rate of complications in the reviewed studies [34]. These findings suggest that topical NSAIDs may be a reasonable short-term option for pain control in uncomplicated corneal abrasions. Ketorolac is among the most commonly prescribed agents and is typically administered four times daily for up to three days or until epithelial healing, as short-term use is supported by available evidence and longer courses have not demonstrated additional benefit [26,34]. Patients frequently report transient stinging upon instillation. Prolonged or excessive use has been associated with rare but serious adverse effects, including corneal melt, underscoring the importance of limiting duration and monitoring for toxicity.

Topical Anesthetics

The use of topical anesthetics for pain control in corneal abrasions remains controversial, reflecting differing perspectives between emergency medicine and ophthalmology [35]. While short-term pain relief has been observed, concerns persist regarding epithelial toxicity, delayed healing, and misuse [36]. Systematic reviews have found insufficient evidence to support routine outpatient use, particularly given limited follow-up in available trials [35-37]. Overall, available data do not clearly demonstrate a sustained benefit that outweighs potential risks.

In February 2024, the American College of Emergency Physicians (ACEP) issued clinical guidance supporting the limited outpatient dispensing of topical anesthetics (approximately 1.5-2 mL) for selected patients with uncomplicated corneal abrasions, to be used every 30 minutes as needed for up to 24 hours [35]. This recommendation emphasizes strict dosing limits, patient education, and short duration of use to improve acute pain control and reduce opioid exposure.

In contrast, the American Academy of Ophthalmology (AAO) did not endorse these recommendations, citing concerns about real-world feasibility, patient adherence, and the potential for serious complications if dosing limits are exceeded [35]. Ophthalmology literature documents cases of toxic keratopathy associated with frequent dosing or prolonged use beyond 24 hours, including persistent epithelial defects, stromal inflammation, corneal melt, and perforation, which may result in permanent vision loss [27,35,36].

Evidence Limitations and Clinical Implications

The evidence base supporting outpatient topical anesthetic use remains limited. Available randomized trials are small, heterogeneous in design, and often include short follow-up periods that may not capture delayed toxicity or misuse [35,36]. A 2023 Cochrane review concluded that the certainty of evidence is very low and insufficient to support routine outpatient use; although short-term pain reduction was observed in some comparisons, concerns remained regarding delayed epithelial healing and incomplete safety assessment beyond early follow-up [36].

A key concern is that outpatient access may increase the likelihood of repeated dosing beyond intended limits, particularly in patients with severe pain or limited access to follow-up care. In practice, commercially available vials may contain more drops than required for a 24-hour course, and adherence to disposal instructions cannot be reliably ensured [35,37]. For these reasons, routine outpatient prescribing remains controversial.

Given the low certainty of benefit beyond short-term analgesia and the potential for serious harm, a cautious approach is warranted. When pain control is required, topical NSAIDs and oral analgesics have more consistent evidence for symptom reduction without comparable toxicity concerns [34]. Further studies with standardized abrasion classification, validated pain outcomes, and longer ophthalmologic follow-up are needed to better define whether a subset of patients may safely benefit from limited outpatient topical anesthetic use [35].

Bandage Contact Lenses

BCLs reduce friction between the eyelid and exposed corneal nerves and may improve comfort and epithelial healing [25,38,39]. They are commonly used after corneal surgery and have gained popularity as an alternative to pressure patching. In practice, BCL placement is typically performed by ophthalmologists or optometrists trained in contact lens fitting, rather than by primary care or emergency clinicians. Their use is generally reserved for large, painful, noninfectious epithelial defects, such as traumatic abrasions or recurrent corneal erosions, and is typically avoided in contact lens-related abrasions or when infectious keratitis is suspected due to increased infection risk [26,38]. However, infection risk limits their use in patients with poor follow-up or high susceptibility to microbial keratitis [38].

Cycloplegic Agents

Cycloplegics may provide symptomatic relief in larger or more painful abrasions by reducing ciliary spasm [22]. When used, a short-acting agent such as cyclopentolate may be administered as a single dose or for a brief course (typically 24-48 hours), as prolonged use is rarely necessary once acute pain improves [22,26].

Follow-Up and Referral

Most uncomplicated corneal abrasions heal within 24-72 hours; however, follow-up should be individualized based on injury characteristics and patient risk factors [26]. Small abrasions with normal baseline vision and improving symptoms may not require routine ophthalmologic follow-up, whereas contact lens-associated abrasions, large epithelial defects, involvement of the visual axis, or injuries related to contaminated or organic material warrant closer follow-up, typically within 24 hours, due to the increased risk of microbial keratitis and delayed healing [26,30]. Immediate ophthalmologic evaluation is recommended for suspected infectious keratitis or corneal ulceration, penetrating injury, retained intraocular foreign body, hyphema, significant vision loss relative to baseline, or worsening pain, redness, or photophobia despite initial therapy [22,26]. Clear return precautions are essential, particularly for patients managed outside ophthalmology settings. Follow-up with an optometrist or ophthalmologist also provides an important opportunity for patient education, including reinforcement of contact lens hygiene, identification of modifiable risk factors, and counseling on protective eyewear and symptom monitoring. This visit allows clinicians to confirm epithelial healing, reassess visual acuity relative to baseline, and address behaviors that may predispose patients to recurrent injury or infection.

Patient Education

Preventive strategies play a key role in reducing corneal abrasion risk. Patients engaged in high-risk activities should use protective eyewear that meets American Society for Testing and Materials standards and is made from polycarbonate material [40]. Contact lens users should receive education on proper hygiene practices to reduce the risk of abrasion and infection.

## Conclusions

Effective diagnosis and management of acute ophthalmologic conditions rely on collaboration and clear communication among healthcare providers. Interprofessional care should integrate patient-centered principles with the best available evidence to ensure safe and consistent clinical decision-making. This review summarizes the pathophysiology, clinical evaluation, and evidence-based management of corneal abrasions, while highlighting areas of practice variability and ongoing controversy. Recent systematic reviews, including Cochrane analyses of topical anesthetic drops, underscore the importance of prioritizing patient safety when evidence for benefit is limited or uncertain. Until larger, methodologically robust studies with standardized outcomes and appropriate ophthalmologic follow-up are available, practices that carry potential risk and lack strong supporting evidence warrant cautious reconsideration.
